# Smartphone-Based Endoscope System for Advanced Point-of-Care Diagnostics: Feasibility Study

**DOI:** 10.2196/mhealth.7232

**Published:** 2017-07-27

**Authors:** Jung Kweon Bae, Andrey Vavilin, Joon S You, Hyeongeun Kim, Seon Young Ryu, Jeong Hun Jang, Woonggyu Jung

**Affiliations:** ^1^ Ulsan National Institute of Science and Technology Department of Biomedical Engineering Ulsan Republic Of Korea; ^2^ Pohang University of Science and Technology Creative IT Engineering Pohang Republic Of Korea; ^3^ Ajou University Hospital Department of Otorhinolaryngology Suwon Republic Of Korea

**Keywords:** smartphone-based endoscope, point-of-care systems, mobile health, low-resource settings

## Abstract

**Background:**

Endoscopic technique is often applied for the diagnosis of diseases affecting internal organs and image-guidance of surgical procedures. Although the endoscope has become an indispensable tool in the clinic, its utility has been limited to medical offices or operating rooms because of the large size of its ancillary devices. In addition, the basic design and imaging capability of the system have remained relatively unchanged for decades.

**Objective:**

The objective of this study was to develop a smartphone-based endoscope system capable of advanced endoscopic functionalities in a compact size and at an affordable cost and to demonstrate its feasibility of point-of-care through human subject imaging.

**Methods:**

We developed and designed to set up a smartphone-based endoscope system, incorporating a portable light source, relay-lens, custom adapter, and homebuilt Android app. We attached three different types of existing rigid or flexible endoscopic probes to our system and captured the endoscopic images using the homebuilt app. Both smartphone-based endoscope system and commercialized clinical endoscope system were utilized to compare the imaging quality and performance. Connecting the head-mounted display (HMD) wirelessly, the smartphone-based endoscope system could superimpose an endoscopic image to real-world view.

**Results:**

A total of 15 volunteers who were accepted into our study were captured using our smartphone-based endoscope system, as well as the commercialized clinical endoscope system. It was found that the imaging performance of our device had acceptable quality compared with that of the conventional endoscope system in the clinical setting. In addition, images captured from the HMD used in the smartphone-based endoscope system improved eye-hand coordination between the manipulating site and the smartphone screen, which in turn reduced spatial disorientation.

**Conclusions:**

The performance of our endoscope system was evaluated against a commercial system in routine otolaryngology examinations. We also demonstrated and evaluated the feasibility of conducting endoscopic procedures through a custom HMD.

## Introduction

Over the past decade, the endoscope has become an essential medical instrument that enables physicians to access the internal organs with magnified visualization. Endoscopy was mainly purposed to investigate the unusual symptoms associated with the interior of the body, but now it has been utilized in a host of medical procedures that assist not only in the diagnosis, but also in the staging of diseases, biopsy, local therapy, and minimally invasive surgery [1-3]. The endoscope finds application in numerous fields, including gastroenterology, orthopedics, urology, obstetrics/gynecology, otolaryngology, neurology, and anesthesiology [4-6]. Currently, the endoscope system incorporates advanced technologies to provide enhanced diagnosis by offering high-definition, fast, quantitative, and wide-viewing images. Despite the multiple benefits of using endoscopy, the routine endoscopic procedures are usually confined to clinics [7]. Moreover, most of the endoscope system itself is not suitable for remote consultation and point-of-care (POC) diagnosis at developing areas or in low-resource settings [8,9].

Recently, there have been efforts to integrate smartphones into various medical devices [4,10-18]. Because of the recent advances in mobile computing electronics, the performance of smartphones is now comparable to personal computers while also featuring the state-of-the-art engineering components such as microsensors, high-resolution camera, powerful central processing unit, graphics processing unit, and high-speed data communication chips [10,18]. The infrastructure of mobile data communications has now become fast and ubiquitous, equipped to efficiently deliver the data between remote sites. Thus, the technical integration between information and communication technology and medical devices has opened a new opportunity for the next-generation diagnostic protocols through telemedicine and big medical data analysis.

In this study, we introduce a complete smartphone-based endoscope system and evaluate its feasibility for clinical applications. Although smartphone technology has already been applied to endoscopic devices, previous works showed only proof of concept while not considering its diagnostic value as well as the user interface. Furthermore, previously developed smartphone-based endoscope devices utilized the built-in camera software in the smartphone that only provided limited functionalities for the endoscopic imaging [7,19]. In this study, we present a full smartphone-based endoscope system combined with the custom hardware/software, light source, as well as a head-mounted display (HMD) for quick office procedures and remote care settings. The performance of this newly developed endoscopic device was compared with one of the commercial endoscope system.

## Methods

### Endoscope System Using Smartphone

**Figure 1:**
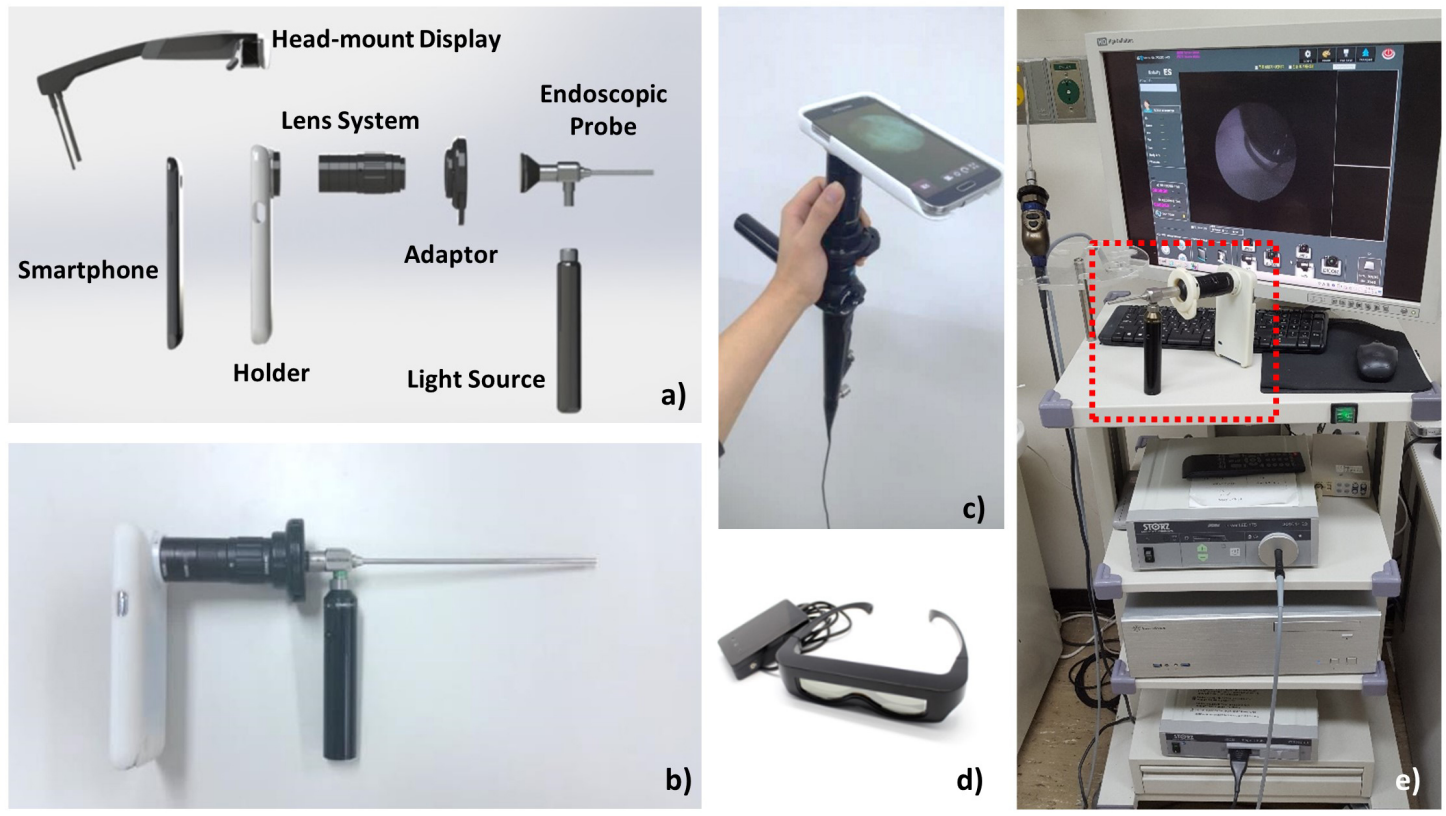
Smartphone-based endoscope system setup. (a) Schematic of smartphone-based endoscope system. A photography of (b) rigid type endoscopic system, (c) flexible type endoscopic system, (d) android based HMD, and (e) the smartphone-based endoscope system vs clinical endoscope system.

**Figure 2:**
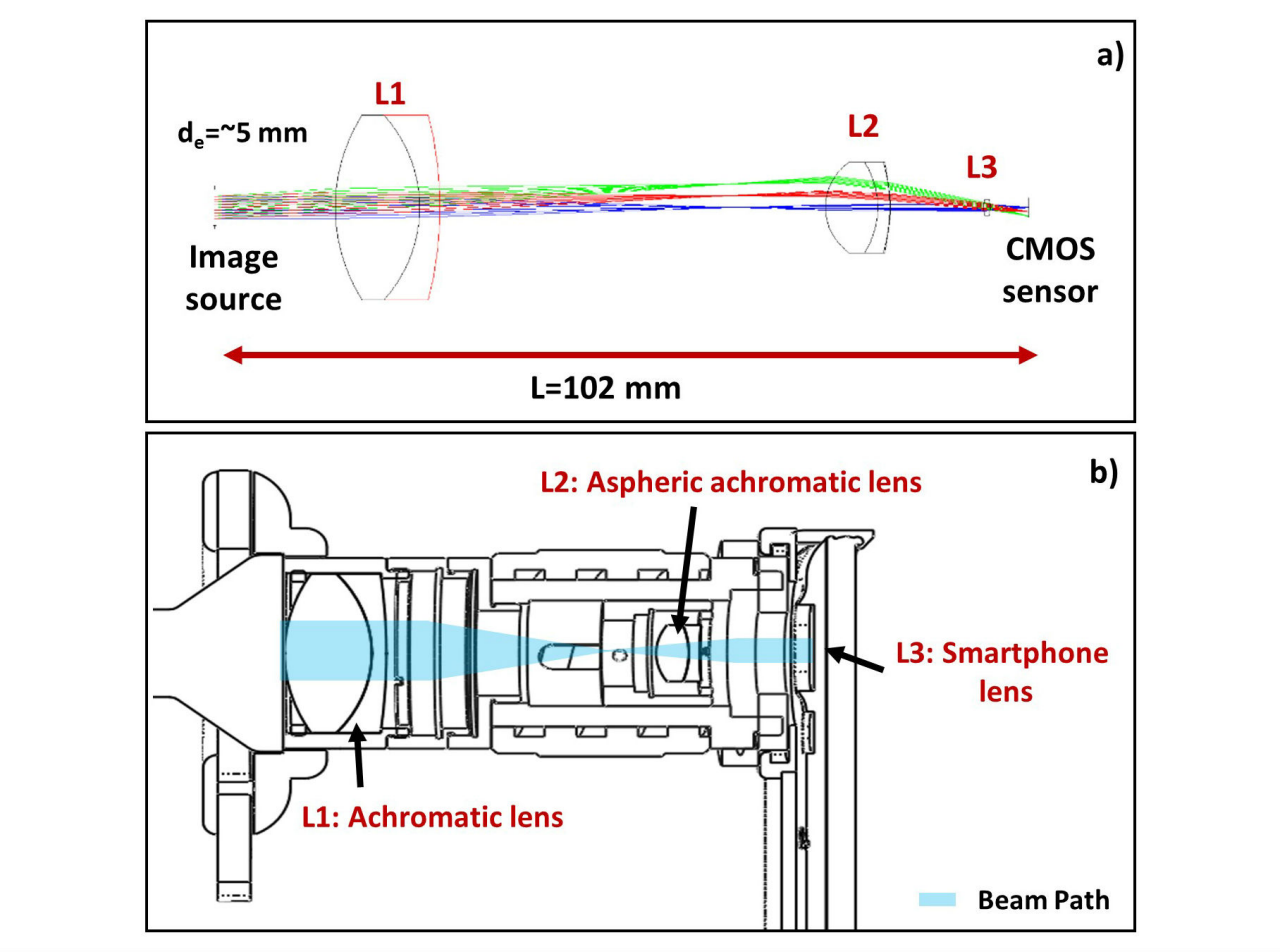
Lens simulation of smartphone-based endoscope system. (a) Ray-tracing of optics for smartphone-based endoscope system. d_e_ denotes a diameter of the eyepiece of endoscope probe, which is the image source. Achromatic lens (L1) having f=40 mm, Ø25.4 mm and aspherized achromatic lens (L2) having f=14 mm, Ø12.7 mm were utilized. Smartphone lens (L3) was simulated to deliver the image to complementary metal-oxide semiconductor sensor. (b) Schematic of assembled lens system in smartphone-based endoscope system.

We developed a low-cost and portable endoscope based on a smartphone as illustrated in Figure 1. Our system comprised 6 pieces of components aligning in series, including a conventional endoscopic probe, compact light source, customized adapter, magnifying relay-lens, packaging holder, and smartphone. Both holder and adapter were designed by three-dimensional (3D) modeling software (Solid Works) reflecting the actual dimensions of the smartphone and the endoscope eyepiece. The endoscopic probe can be detached from the adapter, which is compatible to most of the commercial rigid and flexible endoscopes. For delivering light to the interiors of body cavities, a portable light source (Medit, SPARK) was attached to the illumination port of an endoscopic probe. The smartphone holder was built for a specific smartphone model (Samsung Electronics, Galaxy S5) and made by a 3D printer (Stratasys, Objet260 Connex2) with the spatial precision of ±16 μm. For the magnification of endoscopic image, we customized the lens system that was located between the eyepiece of endoscope probe and smartphone camera. The customized lens system enabled the manual focus by employing the zoom housing (Thorlabs Inc, SM1NR05) to correspond to the different endoscopic probe. Off-the-shelf lenses were designed and selected by the optics designing software (ZEMAX LLC, Optic Studio) as shown in Figure 2. A smartphone camera–mimicking lens (L3) was simulated with 4.8 mm, f/2.2 as a constant parameter, which was set to infinite-focus. To deliver the image to the camera, we utilized 2 achromatic lenses (L1, L2) to reduce the chromatic aberrations while sustaining the portability as well as cost-effectiveness. Thus, tradeoff between length of the lens system and magnification was optimized. We assembled the achromatic lens and the aspherized achromatic lens with 40 mm and 14 mm focal lengths, respectively, to achieve approximately 4× optical magnification, which could be further enhanced up to 12× magnification approximately via software-based digital zoom. The final image was captured by the complementary metal–oxide–semiconductor sensor of the smartphone with specifications of 16.0-megapixels, MP (5312 × 2988 pixels), 1/2.6 inches (1.12 μm pixel size), incorporating 4.8 mm focal length and f/2.2 optical lens.

For replacement of the conventional display and enhancement of portability, a newly designed commercialized HMD module (Green Optics Co, GO Glass) was employed in our system (Figure 1). The operation of HMD was based on the Android operating system which allowed viewing the live endoscopic images through the glasses with 50% transparency, 45-degree sight angle, and 114 × 22 × 14 mm viewing optics. In addition, HMD was able to project the images that used resolution of extended graphics array (1024 × 768) in front of the eyes. The HMD was interfaced wirelessly with the smartphone via a mirror casting technology and displayed the endoscope images to clinicians. As shown in Figure 1, a clinician would attach either the rigid or the flexible endoscope probe to the system and wear an HMD on the head while manipulating the smartphone-based endoscope system.

### User Interface Software Development

**Figure 3:**
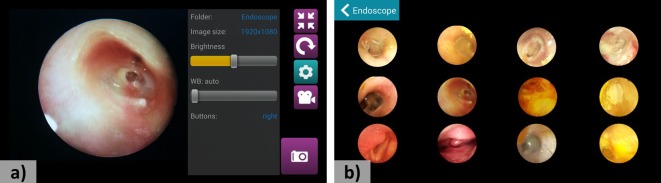
Android based software for the smartphone-based endoscope system. (a) Main capturing screen. (b) Gallery mode of the software.

We developed a JAVA-based Android software for a simple and intuitive user interface that allowed an operator to control essential imaging parameters such as display brightness, image resolution, digital zoom, and focusing adjustment (Figure 3). We also added multiple user-friendly features. For example, user could be set for either left- or right-handed operation for easier control of the display parameters during endoscope manipulation. Displayed endoscopic images could be automatically flipped for better coordination between the endoscope movement and the display orientation. Furthermore, captured images and videos could be visualized in comprehensive gallery mode. For convenient operation of the system, the software was also enabled for simultaneous wireless casting on HMD.

### In-Vivo Imaging Protocol at Otolaryngology

In accordance with a protocol approved by the institutional review board of the Kyungpook National University Hospital, the smartphone-based endoscope system was tested in human subjects. Each imaging session was conducted immediately after subjects’ routine endoscopic examinations at an ENT (ear, nose, and throat) doctor’s office in Kyungpook National University Hospital. Routine endoscope procedures performed in the ENT departments were followed while utilizing our system as well. Before performing the endoscopy, the system was adjusted to get a fine focus for clear images through software and optics. Then, endoscope probe was wiped with a disinfectant to avoid the cross-contamination between patients. The whole procedure took less than a minute, thus avoiding undue burden on volunteers. Volunteer patients were verbally introduced to the risks and goals of this research study and agreed to participate and released the right to their endoscopic data to be used for the publication. For this research, 50 patients aged 20 or older were asked for participation, and of these, 30 volunteers agreed to participate in this research. As this was an initial pilot clinical study, we did not attempt to achieve statistical significance in data.

As it is shown in Figure 1, each human subject was imaged using both smartphone-based endoscope and commercial endoscope system that comprised a charge-coupled device camera head (Stryker, 1188HD), light source (Stryker, X8000), and information management system (Stryker, SDC ULTRA). Various types of endoscope probes such as 0°, Ø4, 50 mm length rigid otoscope (Medstar, Otoscope), 0°, Ø4, 175 mm sinuscope (Medsatar, Sinuscope), 70°, and Ø3.4, 300 mm flexible endoscope (Olympus, ENF-P4) were utilized.

Then, we chose several clear representative endoscopic pictures, including the healthy and diseased models corresponding to each endoscopic probe. The entire process was observed via the smartphone’s liquid crystal display screen as well as the HMD in real time. In addition, the images from the commercial endoscope system were captured and archived via a computer for additional analysis. Thereafter, all captured images were cropped for resizing to better visualize the image for the manuscript. Moreover, the brightness and light contrast were manually adjusted for each image to enhance the visibility. No other image process was implemented in this study.

## Results

### System Characterization

**Figure 4:**
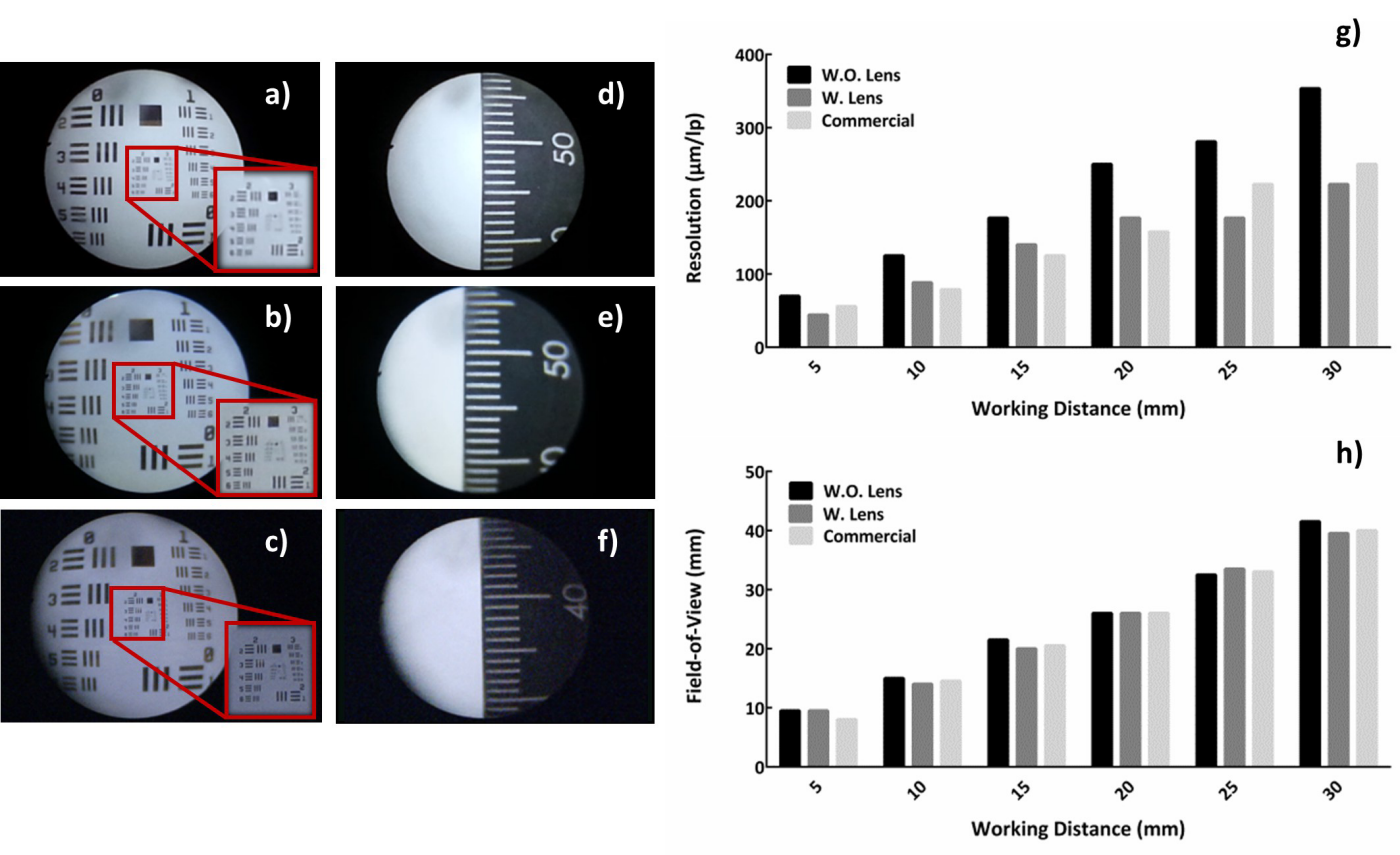
Result of the smartphone-based endoscope evaluation. At 20 mm away from the target, the USAF resolution was captured with (a) smartphone-based endoscope without lens, (b) with customized lens, and (c) commercial endoscope system. At the corresponding distance, field-of-view was measured by capturing the ruler with (d) smartphone-based endoscope without lens, (e) with customized lens, and (f) commercial endoscope system. (g) Graph of the measured resolution, and (h) field-of-view according to the working distance.

To evaluate the imaging performance of our system, the US Air Force resolution target was imaged as shown in left side of the Figure 4 [7,20]. Three different images were acquired and compared with smartphone-based endoscope with/without lens and the clinical version of commercial endoscope system. The distance between the end of endoscope probe and resolution target was 20 mm, which was sufficient to preserve the optical field-of-view (FOV).

Image captured by the smartphone-based endoscope without lens showed the lowest imaging capability in terms of resolution. It was able to resolve 4.0 line pairs per millimeter (lp/mm), which corresponds to 250.0 μm. When custom lens system was applied, the imaging performance improved and was able to resolve 5.66 lp/mm (corresponds to 176.7 μm). The clinical version of endoscope system, showed the highest imaging performance among the 3 systems by resolving 6.35 lp/mm (corresponds to 157.5 μm).

In addition, a ruler was captured to identify the FOV as shown in Figure 4. Similar to the resolution measurement, images were captured at 20 mm of working distance using our device with/without lens and the clinical version of endoscope system. Measurements of resolution and FOV at the working distance between 5 mm and 30 mm can be seen in right side of the Figure 4, respectively. They show that the smartphone-based endoscope with lens has imaging capability similar to that of a clinical endoscope system. Moreover, it can be ascertained that the features of the lens system did not critically influence the FOV.

**Figure 5:**
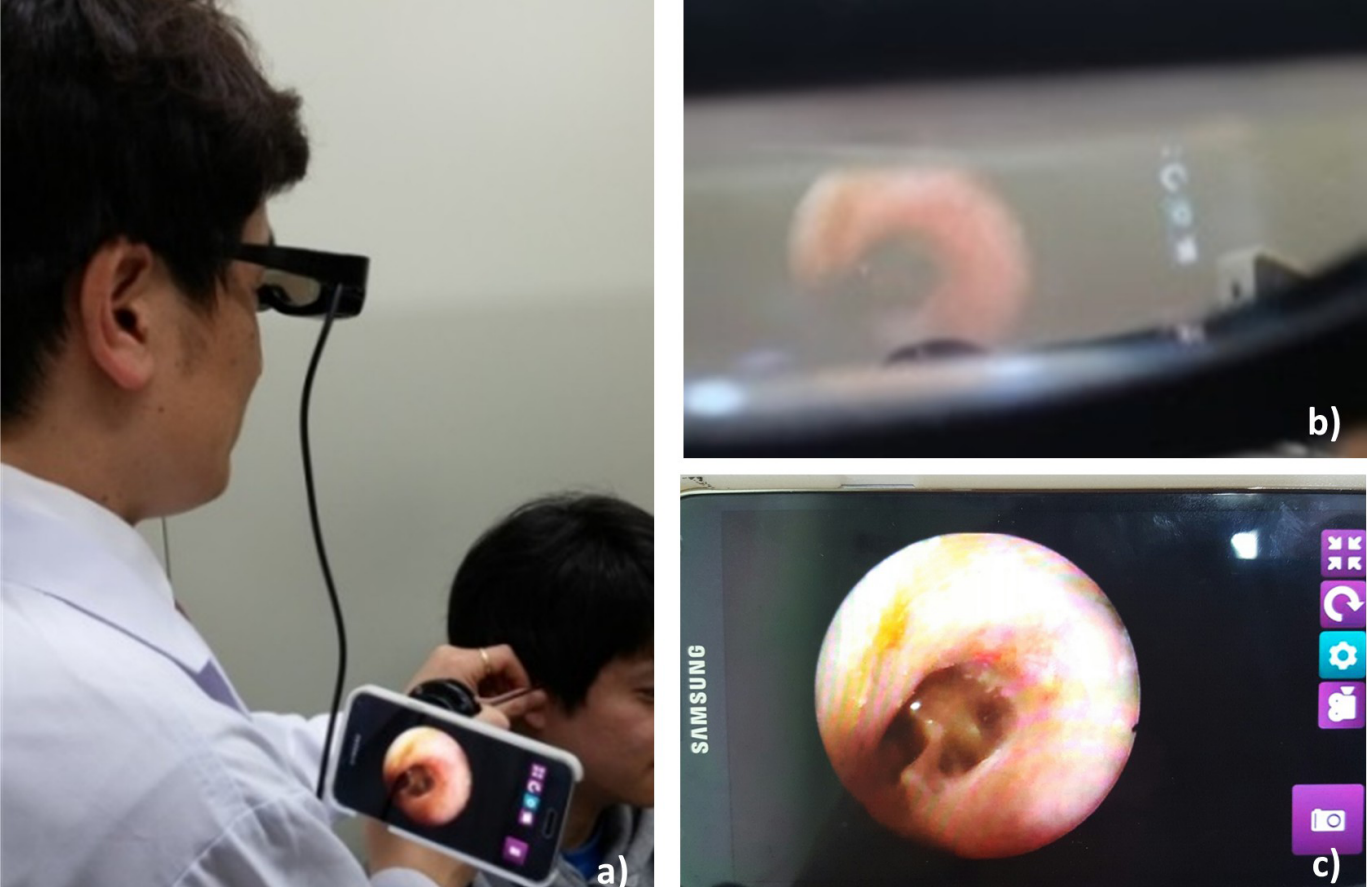
HMD integrated smartphone-based endoscope. (a) A demonstration of the smartphone-based endoscope system using the HMD. (b) Duplicated image shown in viewing optics of HMD. (c) Live endoscopic image shown in the smartphone screen.

### HMD-Based Endoscopy Performance

Routine endoscopic procedure can sometimes be cumbersome for the clinician because of mismatched eye-hand coordination. Manipulating the endoscope probe while observing the monitor forces a divergence between site of manipulating hand and FOV, which requires extensive training. Moreover, clinicians easily experience ocular strain from endoscopic procedures. To overcome the abovementioned issues, HMD was applied to endoscopic procedures [20-23]. We tested the feasibility of endoscopic diagnosis using our device, which included a see-through type of HMD as shown in Figure 5. Images were acquired with 3264 × 2176 (8MP), International Organization for Standardization (ISO) 100, lowest exposure (−4) settings using the smartphone-based endoscope in routine endoscope procedure. The Android-based HMD controller successfully duplicated the screen of the smartphone; hence, images could be seen through the viewing optics in the HMD (b, c). The clinician was able to detect the lesions in front of the eyes and successfully manipulated the endoscopic probe. Moreover, through this system setting, the clinician had a benefit of observing the endoscopic image simultaneously at the manipulation site, thereby reducing spatial disorientation. The use of HMD during the endoscopic procedure showed a potential of improvement on the eye-hand coordination. The clinician experienced a reduction in diagnosis time as compared with observing through the monitor. In addition, it was found that the manipulation time for flexible endoscope probe was significantly reduced when clinician wore an HMD.

### In-Vivo Human Subject Imaging

The smartphone-based endoscope was tested in a routine otolaryngology examination to evaluate its clinical value and performance. The images of human subjects were captured and compared with a clinical endoscope system. Our device used 3264 × 2176 (8MP) image resolution at the automatic ISO mode (ISO 100), and the lowest exposure (−4) setting to suppress the saturation of light and to achieve clear images. For the clinical endoscope system, the 1280 × 1024 (1.3MP) was used for camera resolution settings. The images were obtained at the same location with the corresponding probes that were utilized in the commercial system.

**Figure 6:**
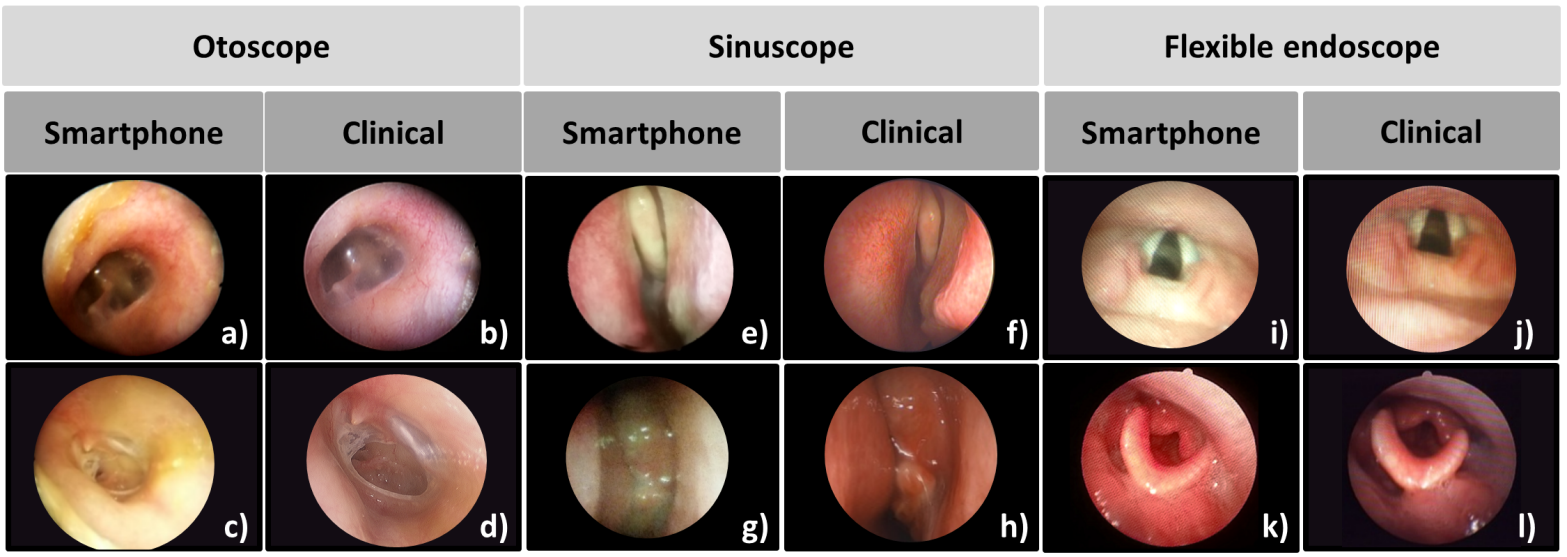
Result of human in-vivo imaging at Otolaryngology. Otoscope, sinuscope, and flexible endoscope were utilized. The corresponding sites were captured with smartphone-based endoscope and commercial endoscope system in clinical settings. (a, b) normal tympanic membrane. (c, d) chronic otitis media, central large perforation. (e, f) normal middle turbinate. (g, h) chronic rhinosinusitis with nasal polyp. (i,j) normal vocal cord. (k,l) postoperative state of thyroid cancer surgery in larynx.

Images captured with the smartphone-based endoscope are shown in the columns on the left side, whereas images acquired from the clinical endoscope system are shown in the columns on the right under each subcategory of endoscopic probes (Figure 6). To examine different areas in otolaryngology, different types of endoscope probes were applied. Images of tympanic membranes were captured using the otoscope probe (the 1st two columns in Figure 6). Images from both systems clearly showed the structures of the tympanic membrane compared with that of the normal patient. For nasal examination, a sinuscope was applied (2nd and 3rd coulmns in Figure 6). Several images were captured during the endoscopic examination to observe the feasibility of our system. Among the nasal endoscopic images, both systems particularly highlighted the nasal polyps of patient with chronic rhinosinusitis (g, h). The flexible endoscope was also applied to examine the larynx (the last two columns in Figure 6). Flexible endoscope was inserted first through the nose and then through nasopharynx to reach the larynx of the patients. Some images obtained by the fiber-optic endoscope probe displayed the pixelation throughout the entire FOV affecting the image quality and limiting the spatial resolution by undersampling because of core-to-core spacing between fibers [24]. Despite the pixelation, the smartphone-based endoscope still provided sufficient contrast and image quality for detecting the lesions. The smartphone system showed an acceptable level of clarity for an ENT specialist to distinguish the healthy and the diseased or damaged tissue regions through the smartphone screen.

**Table 1:** Cost of smartphone-based endoscope system.

Components	Price in US dollars
Lens system (2 off-the-shelf lenses, adjustable lens tube, additional lens components)	~$ 400
Three-dimensional printing parts (smartphone case, adapter, and coupler)	~$ 50
External light source	~$ 300
Miscellaneous components (spring, screws, and battery)	~$ 30
Smartphone (Samsung Galaxy S5 16GB)	~$ 200
Total	~$980

## Discussion

### Principal Findings

In this study, we demonstrated the use of a novel endoscope system based on a smartphone in clinical applications. The benefits of various aspects of our device have been observed throughout the study. Our device was fabricated in a compact form factor, so it can be used as a carry-on or standalone medical device in a clinical environment. Furthermore, significant safety guards or chemical protection were not necessary for this device. However, additional precaution was taken by wiping the probe with an antiseptic fluid to avoid the cross-contamination. The 3D printed case offered the robustness as well as the reliability with familiar coupling mechanism compared with that of the conventional system for endoscope probe. It was much simpler to set up the device for endoscopic diagnostics. The use of smartphone for endoscopy further simplified the system by providing the state-of-the-art sensors and hardware, thereby eliminating the need for expensive ancillary devices [10,18]. In addition, cumbersome image processing or device control algorithm was unnecessary for endoscopic diagnosis with smartphone. Thus, the smartphone-based endoscope significantly reduced the overall cost compared with the price of conventional endoscope system equipped with essential apparatuses. The average price of the conventional system, with our survey from three major endoscope companies, was over US $ 50,000, whereas our system only costs under US $1000, including the smartphone (Samsung Galaxy S5) and the endoscopic probe. Even the additional cost related to commercial product delivery were taken into account, making the estimated price substantially lower for the smartphone-based system. Table 1 clearly presents the prices of components in our smartphone-based endoscope system.

Unlike other devices available in the market, the smartphone-based endoscope presented in this study provides a full endoscopic capability with a potentially customizable endoscope platform. Rather than simply capturing endoscope images with enhanced mobility, our system has shown a possibility to provide more advanced imaging modalities with various contents to the field applications. It can be easily modified for various needs in every aspect (optics, 3D printed cases, and light source) or made compatible with various accessories to the system for expanding the functionalities. We demonstrated the HMD as an example of a seamless integration of an accessory for endoscopy procedure. The integration of the next-generation wearable display such as an HMD can provide several advantages to the smartphone-based endoscope system. An HMD allows clinicians to monitor digital information that is superimposed over the real-world view, thereby eliminating the need to view a monitor away from the patient and reducing the discomfort in the working position. Furthermore, the HMD completely rendered the smartphone-based endoscope system more portable and compact. However, some clinicians have experienced dizziness when they utilized an HMD for a long time because of ghost images caused by inherent structure of HMD design (static distance between optical windows). We anticipate that the future model of HMD will address these limitations and provide more advantages in our system.

The results shown in this study demonstrate the potential of the smartphone-based endoscopy for various clinical applications outside the conventional ones. In particular, the HMD-integrated system may offer more benefits outside the clinic. Faster diagnostics may be enabled with fascinating ease and safety, with considerable reduction in mismatch of the eye-hand coordination through HMD. The linkage between biosensors and HMD-integrated smartphone-based endoscope system could be beneficial for endoscopic surgeries. The extremely low price of the system could make it a cost-effective education tool for endoscope training. Furthermore, ubiquitous telecommunication infrastructure enables smartphone-based endoscope to be useful even in medical emergencies in the ambulance or field hospitals. It is still difficult to arrange an emergency operation in a timely manner. For this reason, sending the diagnosed endoscopic images to specialists while transporting the patient could shorten the delays and help manage urgent situations more efficiently using the smartphone-based endoscope.

Throughout the experiments, the great potential of the integration of smartphone technologies with medical devices has been observed because of the tremendous reduction in costs while sustaining health benefits for the patients. Many literatures have described them as the most prospective devices for the next-generation POC diagnostics [18,25-28]. The smartphone-based medical devices may complement the quality of the services such as medical and health informatics. Moreover, recently developed mobile-picture archiving and communication system allows to connect remote locations to the central patient database in a secure fashion [29-31]. Now, real-time on-site diagnostics are available with a secure transmission of personal and sensitive medical data in a format specific to the health care industry [29]. These mobile devices show the possibilities of providing assistance to the patients in their medical records to improve coordination among health care providers who use telecommunication technologies such as short message service, calls, and Internet-based video links [18,25,27]. With advances in technologies, many devices tested in the laboratory settings have been translated to clinically or commercially available products [18,25-28]. The smartphone-based medical devices, especially for imaging device such as endoscope, will soon be utilized in the surgical settings for providing reliable results.

### Conclusions

Recent advances in smartphone technology have enabled the realization of cost-effective, portable medical devices. In this study, we introduced an endoscope system using a smartphone as an imaging sensor and display suitable for the POC diagnostics and offering the unique advantages such as mobility and flexibility. Experimental results showed that our device could potentially provide sufficient imaging performance as a diagnostic tool in a wide range of nonclinical settings and ultimately, in some clinical settings. In particular, our device would be a very useful tool for health providers in low-resource settings and at remote locations with limited health care service.

## Abbreviations

ENT: ear, nose, and throat
FOV: field-of-view
HMD: head-mounted display
ISO: International Organization of Standardization
Lp/mm: line pairs per millimeter
MP: megapixels
POC: point-of-care
3D: three-dimensional
